# Knowledge and attitude of Uyghur women in Xinjiang province of China related to the prevention and early detection of cervical cancer

**DOI:** 10.1186/s12957-015-0531-8

**Published:** 2015-03-14

**Authors:** Abida Abudukadeer, Sumeyya Azam, Ayi Zuoremu Mutailipu, Liu Qun, Guo Guilin, Sayipujiamali Mijiti

**Affiliations:** Department of Gynaecology, First Affiliated Hospital of Xinjiang Medical University, 830054 No. 393 Xin Yi road, Urumqi, Xinjiang People’s Republic of China

**Keywords:** Cervical cancer, Human papillomavirus, Human papillomavirus vaccine

## Abstract

**Background:**

Cervical cancer is one of the commonest causes of cancer-related deaths worldwide. The prevalence rate of cervical cancer in Uyghur women in Xinjiang Autonomous Region of China has been remarkably higher than the ethnic groups living in the same region. This study aimed to assess the knowledge level and attitude of cervical cancer and its issues among the Uyghur women in Xinjiang province of China.

**Methods:**

A cross-sectional interview-based survey of 5,000 Uyghur women was developed from 2013 to 2014 in Xinjiang autonomous region, to assess their knowledge and perception of cervical cancer and its issues. The collection of data was based on the questionnaire items.

**Results:**

According to the questionnaire items, we collected a data for 5,000 participants. A very small proportion of participants had heard of the cervical cancer, human papillomavirus (HPV), and HPV vaccine, that is, 27.0%, 13.0% and, 6.0%, respectively. According to the demographic characteristics, women aged 31 to 40 years were more aware of the knowledge of cervical cancer (32.9%), HPV (17.8%), and HPV vaccine (9.1%), and women with undergraduate or higher educational level had more knowledge of cervical cancer (30.0%), HPV (21.0%), and HPV vaccine (9.7%).

**Conclusions:**

From our study, we concluded that Uyghur women need more information about cervical cancer and its risk factors. Lack of the related knowledge about cervical cancer may be one of the important factors for high incidence rate of cervical cancer in Uyghur population. In order to reduce the incidence rate and mortality of cervical cancer in Uyghur women and to make extensive health education to raise awareness of cervical cancer and HPV is strongly needed than prophylactic vaccination.

## Background

Cervical cancer continues to be one of the leading female genital cancers worldwide. About 80% of cases occur in developing countries and about 87% cervical cancer deaths occur in less developed regions [1-3]. China accounts for 29.0% of the 51,000,000 new cases of cervical cancer each year [4]. In China, there is an annual incidence of about 46,000 cases, and cervical cancer presents a major health problem [5].

Xinjiang province has the highest incidence (590/100,000) and mortality rate of cervical cancer in China, especially the south of Xinjiang. The incidence of cervical cancer among the Uyghur women is four times higher than the mean incidence of China (138/100,000) [6,7]. The prevalence of cervical cancer in Uyghur women has been remarkably higher than the ethnic groups living in the same region, and the mortality rate was more than the other ethnic minorities in China.

Epidemiologic evidence clearly indicates that high-risk human papillomavirus (HPV) is the principal cause of invasive cervical cancer and cervical intraepithelial neoplasia (CIN). Invasive cervical cancer is attributed to HPV infection. Seventy percent of the cancers are known to be caused by HPV 16 or 18 [8].

According to our previous study, HPV 16 was the most common type detected in Uyghur women with squamous cell carcinoma and CIN in Xinjiang. The prevalence of HPV 18 and 58 was relatively low, and all other types were absent. Together with the high infection rate, this may be the reason for the fourfold higher cervical cancer incidence in this province and in this population [9].

Cervical cancer is a deadly disease once it reaches the invasive stages, but out of all the female genital tract cancers, it is the only preventable cancer if detected at its early stages [10]. Therefore, the key to reducing cancer morbidity and mortality is still the early detection and treatment of pre-cancerous lesions.

Cervical cancer can be effectively controlled through primary and secondary prevention such as, cervical screening and prophylactic HPV vaccination. Since the Pap smear test was introduced for routine screening, a substantial decline has been witnessed in cervical cancer deaths in developed countries in the last four decades [11].

It is a general consensus that the cytology screening for cervical cancer is effective in reducing the incidence and mortality in developed countries. In contrast to this striking result, cervical cancer is the second most common cancer among women and the leading cause of cancer death in developing countries due to inadequate use of the screening service.

HPV vaccines are expected to effectively avert cervical morbidity and mortality in China, if it was routinely used in women. But because of the limitations of prophylactic HPV vaccination, it is imposing a substantial burden to the women health in the developing countries. Therefore, evaluate the awareness and knowledge of screening for cervical cancer among the high risk people and to make good education program is vital to reduce the incidence of cervical cancer in developing countries. Various studies have been undertaken to evaluate women’s awareness and knowledge of cervical cancer and screening for cervical cancer [12-14].

With the lack of knowledge about the disease and familiarity with the concept of prevention, there is no way to mention early detection of cervical cancer and use of HPV vaccination.

It is not clear how well HPV and its association to cervical cancer is known in the general population, especially in Xinjiang.

This study was undertaken to explore the knowledge of Uyghur women in Xinjiang Autonomous Region, China, regarding cervical cancer issues, HPV, and HPV vaccine.

## Methods

### Setting

A cross-sectional study was carried out among the Uyghur women in Xinjiang province of China. A total of 5,000 women were recruited in our investigation between 2013 and 2014. The sample population was composed of ≤20 years to ≥51 year olds. A questionnaire with the options of ‘yes’ and ‘no’ for each item was used for the collection of data. A total of 5,625 questionnaires were distributed, and out of them, 5,000 participants responded. The participants were recruited after door-to-door investigation. Face-to-face interviews were administered by the team of doctors from the First Affiliated Hospital of Xinjiang Medical University, China.

### Questionnaire/data collection

The underlying concept and format of the questionnaire was developed by our review of literature and research hypotheses. The questionnaire was designed to assess the knowledge about cervical cancer, HPV, and HPV vaccine by demographic variables and life style factors. The questionnaire was modeled in such a manner that the women could provide answers to the personal questions in private, without requiring assistance of an interviewer.

The listed questionnaire items were also mentioned in Uyghur language to avoid language barrier and to get better response from the respondents.

Prior to the initiation of the survey, practice interviews were conducted in order to ensure that the interviews were undertaken in a standardized manner. The study design was approved by the Ethics Committee of First Affiliated Hospital of Xinjiang Medical University.

### Data analysis

For statistical analysis, SPSS statistics (version 17.0) was used. Chi-squared (*X*^2^) test was used to examine the differences in demographic variables between women with knowledge about cervical cancer, HPV, and HPV vaccine. Logistic regression was used with the inclusion of age and education as the factors in the logistic regression models. *P* value of <0.05 was considered statistically significant.

## Results

A total of 5,625 questionnaires were distributed, and out of them, 5,000 (88.9%) participants responded. The age of the women ranged from ≤20 years to ≥51 year olds.

Table 1 comprises the questionnaire items showing the Uyghur women knowledge about cervical cancer and issues related to it.

**Table 1:** **Uyghur women knowledge and analysis of related elements of cervical cancer, HPV, and HPV vaccine**

**Questionnaire items**	**Uyghur women**	
	**Number**	**Percentage**
Have you heard of cervical cancer?		
Yes	1,350	27
No	3,650	73
From where you heard of cervical cancer?		
Friends	475	35.2
Television	458	33.9
Broadcast	117	8.7
Magazine	90	6.7
Others	210	15.5
Do not know	3,650	73.0
Do you know the need of cervical cancer screening once a year?		
Yes	1,000	20.0
No	4,000	80.0
Do you know the risk factors of cervical cancer?		
Yes	900	18.0
HPV	350	7.0
Having HIV	82	1.64
Smoking	67	1.34
Using birth control pills for a long time (5 or more years)	42	0.84
Having given birth to three or more children	64	1.28
Having several sexual partners	104	2.08
Being younger than 17 at first full term pregnancy	79	1.58
Having a family history of cervical cancer	112	2.24
No	4,100	82.0
Do you want to have regular gynaecological examination?		
Yes	900	18.0
No	4,100	82.0
Why you never had regular gynaecological examination?		
Because of the economy problem	1,681	41.0
Because of lack of awareness of the importance of regular gynaecological examination.	1,804	44.0
Because of lack of time	1,515	30.3
Have you ever had cervical Pap smear test?		
Yes	1,650	33.0
No	3,350	67.0
Do you know the significance of cervical Pap smear test?		
Yes	1,350	27.0
No	3,650	73.0
Have you heard of HPV?		
Yes	650	13.0
No	4,350	87.0
Do you know the link between HPV and cervical cancer?		
Yes	350	7.0
No	4,650	93.0
Have you ever had an HPV test before?		
Yes	450	9.0
No	4,550	91.0
Have you heard of HPV vaccine?		
Yes	300	6.0
No	4,700	94.0
Do you think men could be the risk factor for cervical cancer?		
Yes	1,250	25.0
No	3,750	75.0
Do you think sexually transmitted diseases can be prevented if men use condoms?		
Yes	1,400	28.0
No	3,600	72.0

The women were asked to tell whether or not they had heard of cervical cancer and then asked to provide answers of 13 more questions that served to evaluate the level of cervical cancer and HPV knowledge.

### Cervical cancer knowledge

Overall, very less proportion of participants (1,350 (27.0%)) had heard of cervical cancer. Out of them, 475 (35.2%) responded that they heard of cervical cancer from friends, 458 (33.9%) responded that they heard of cervical cancer through television, 117 (8.7%) responded they had heard of cervical cancer from broadcast, 90 (6.7%) responded that they got to know about cervical cancer from magazines, 210 (15.5%) of the respondents had heard of it by other means, whereas 3,650 (73.0%) participants were unsure about it. A total of 3,650 (73.0%) participants had never heard of cervical cancer. As shown in Table 2, at least 1,385 (27.7%) participants had undergraduate or higher level of education. Women who had undergraduate or higher level of education had more awareness of cervical cancer, that is, 30.0% as compared to the women with lower level of education. So, a significant difference was found in the educational levels of the participants with different levels of knowledge about cervical cancer (*P* < 0.05).

**Table 2:** **Assessment of Uyghur women knowledge by educational level**

	**Participants**		**Aware of cervical cancer**		**Aware of HPV**		**Aware of HPV vaccine**	
	**Number**	**Percentage**	**Number**	**Percentage**	**Number**	**Percentage**	**Number**	**Percentage**
Illiteracy	315	6.3	53	16.8	12	3.8	7	2.2
Primary education	1,000	20.0	247	24.9	71	7.1	35	3.6
Secondary education	1,085	21.7	285	26.3	102	9.4	49	4.5
Specialist/professional education	1,215	24.3	349	28.7	173	14.3	74	6.1
Undergraduate or higher education	1,385	27.7	416	30.0	292	21.0	135	9.7
*P* value			*P* = 0.000		*P* = 0.000		*P* = 0.000	

Linear-by-linear association has been used; the difference was statistically significant *P* < 0.05. With the increase of educational level, awareness has increased.

As shown in Table 3, most of the participants were between the ages of 31 and 40 years. A total of 1665 (33.3%) participants were ranged between the age group of 31–40 years. Out of them 549 (32.9%) participants were aware of cervical cancer. Participants aged ≤20 years were less that is, 330 (6.6%) participants. And out of them only 65 (19.7%) participants knew about cervical cancer. The difference was statistically significant (P < 0.05).

**Table 3:** **Assessment of Uyghur women knowledge according to the age group**

**Group**	**Participants**		**Aware of cervical cancer**		**Aware of HPV**		**Aware of HPV vaccine**	
	**Number**	**Percentage**	**Number**	**Percentage**	**Number**	**Percentage**	**Number**	**Percentage**
≤20 years	330	6.6	65	19.7	11	3.3	8	2.4
21 to 30 years	1,330	26.6	317	23.8	123	9.2	72	5.4
31 to 40 years	1,665	33.3	549	32.9	297	17.8	152	9.1
41 to 50 years	1,165	23.3	302	25.9	170	14.6	46	3.9
≥51 years	510	10.2	117	22.9	49	9.6	22	4.3
*P* value			*P* = 0.000		*P* = 0.000		*P* = 0.000	

Chi-squared (*X* ^2^) test has been used; the difference was statistically significant when *P* < 0.05.

According to the analysis of related elements of cervical cancer, shown in Table 1, the overall knowledge of the risk factors for cervical cancer was generally poor with only 900 (18.0%) women knowing at least one of the risk factors. Out of them, 82 (1.64%) women answered affirmatively that HIV could be one of the risk factors of cervical cancer, 67 (1.34%) participants answered affirmatively for smoking as a risk factor, 42 (0.84%) participants knew that using birth control pills for a long time could be a risk factor for cervical cancer, 64 (1.28%) participants answered ‘yes’ for having given birth to three or more children and, 79 (1.58%) participants answered ‘yes’ for being younger than 17 at first term pregnancy as a risk factor for cervical cancer. Most of the participants answered affirmatively for HPV, having several sexual partners and, having a family history of cervical cancer, that is, 350 (7.0%) participants, 104 (2.08%) participants, and 112 (2.24%) participants, respectively, whereas majority of the study population (4,100 (82.0%)) were unaware of the risk factors of cervical cancer, and only 1,250 (25.0%) participants had an idea that males could be the risk factor for cervical cancer. Next, we asked some general questions regarding all the possible reasons for women that ‘Do you want to have regular gynaecological examination?’ Only 900 (18.0%) participants answered affirmatively, whereas 4,100 (82.0%) participants refused to have regular gynaecological examination. Furthermore, we asked ‘why they never had regular gynaecological examination?’ Of the participants, 1,804 (44.0%) answered that they were unaware of the importance of regular gynaecological examination, 1,681 (41.0%) participants answered that they had financial problems, and 1,515 (30.3%) participants answered that they were having lack of time. Of the study population, 1,650 (33.0%) had and 3,350 (67.0%) had not previously undergone Pap smear tests, and 1,350 (27.0%) women knew the significance of cervical Pap smear tests, whereas 3,650 (73.0%) women were unaware of the significance of cervical Pap smear tests. Among the respondents, only 1,000 (20.0%) women had the awareness of the need of cervical cancer screening once a year, whereas 4,000 (80.0%) women had no awareness of its need.

### HPV knowledge

Overall, only 650 (13.0%) of the participants answered that they had awareness of HPV and out of them 292 (21.0%) participants had undergraduate or higher educational level (Table 2). A large number of the participants, that is, 4,350 (87.0%) participants were totally unaware of HPV. A statistically significant difference was found among the educational levels of the participants with different levels of knowledge of HPV (*P* < 0.05).

As shown in Table 3, according to the different age group women knowledge about HPV, overall 650 (13.0%) women knew about HPV. Most of the participants were between the age of 31 and 40 years. Out of the 1,665 (33.3%) participants between the age group of 31 to 40 years, 297 (17.8%) participants knew about HPV. A statistically significant difference (*P* < 0.05) was identified in age groups relative to the women with different knowledge levels of HPV. Furthermore, only 350 (7.0%) participants knew the link between HPV and cervical cancer, whereas 4,650 (93.0%) participants had no idea about it. Four hundred fifty (9.0%) participants had and 4,550 (91.0%) participant had not previously undergone HPV test.

### Knowledge of HPV vaccine

Overall, only 300 (6.0%) participants had heard of HPV vaccine, whereas 4,700 (90.0%) had never heard of HPV vaccine (Table 1).

According to Table 2, 1,385(27.7%) participants had undergraduate or higher levels of education, which was more than the women who had lower educational level. Only 290 (6.0%) participants had an affirmative answer about their knowledge of HPV vaccine. Participants with undergraduate or higher level of education had more awareness of HPV vaccine, that is, 135 (9.7%). A statistically significant difference was identified among the different educational levels of the women with different knowledge levels about HPV vaccine.

As previously mentioned that most of the participants were between the age of 31 and 40 years (Table 3), 152 (9.1%) participants between this age group had more knowledge of HPV vaccine. The difference was statistically significant (*P* < 0.05).

## Discussion

To our knowledge, this is the largest study that has indicated the awareness of cervical cancer screening and HPV among Uyghur women in Xinjiang, a high-incidence region of cervical cancer in China. Lack of knowledge about cervical cancer and Pap smear, certain demographic factors and unfavourable attitude towards Pap smear test can have negative impact on utilization of the test by women. On the other hand, cervical cancer preventive programs can be effective in increasing cervical cancer knowledge, perceived susceptibility, and cancer prevention behaviour [15].

A cross-sectional study was carried out among the 5,000 Uyghur women in Xinjiang Uyghur Autonomous region, China. The purpose was to evaluate their knowledge regarding cervical cancer, HPV and HPV vaccine. The resultant of this survey was that less than a quarter of the respondents were not at all or very less aware of cervical cancer, HPV, and HPV vaccine.

As foreseeable, awareness was lower in women with lower educational levels. With the increase of educational level, awareness has increased. Earlier studies have shown important association between health behaviour and educational level [16]. Low literacy is associated with low income and poor health status. Cervical cancer mortality rate is markedly higher among illiterate women. Interventions aimed at improving health literacy may result in more functional interaction with the healthcare system and less anxiety related to illness, and it may affect the health patterns of a woman’s family members [17].

Developed countries are far ahead because of both organized and opportunistic Pap screening programs. Screening rate and Pap smear coverage are 88% and 93%, respectively, in the USA [18,19]. Cervical cancer screening rate is 63.9% in South Korea, while 7% in rural India [20], 9.8% in South Africa [21], 17.2% in Sri Lanka [22], 27.1% in Iran [23], and 45.2% in South Turkey [24].

Since the Papanicolaou (Pap) smear test was introduced for routine screening, a substantial decline has been witnessed in cervical cancer deaths in developed countries in the last four decades [25].

A recent survey in Nepal found that only 15.7% of the participants had utilized cervical Pap smear test in the past [26] which is lower than that reported in our survey. In the current study, 33.0% of the participants responded that they had utilized cervical Pap smear test in the past and the knowledge about the need of cancer screening once a year was present only in 20.0% of the respondents. Women’s attitude and practices towards screening were negative. Our survey suggests a strong need to raise public awareness about cervical cancer screening.

Because of the limitations of prophylactic vaccination especially in developing countries, cervical screening test is still a very effective and useful method for reducing high incidence and mortality of cervical cancer in Xinjiang.

In developed countries, cervical screening programs have reduced the incidence of invasive cervical cancer up to 80.0% although this decline has now reached a plateau with current cancers occurring in patients who have failed to attend for screening or where the sensitivity of the tests have proven inadequate.

It is known that precancerous lesions are detectable for a few years or more before cancer develops. If our people have related knowledge and health conception about cervical cancer screening, then this is a long enough golden period and good opportunity to screen and prevent this disease.

In order to control or reduce high incidence of cervical cancer, elevation of knowledge level about cervical cancer is more important than prophylactic vaccination in Xinjiang. It was an important task and project on the prevention and to control the incidence of cervical cancer.

The comparison of the findings of the current study with the other recent surveys is shown in Table 4.

**Table 4:** **Comparison of the current study results with the other recent surveys regarding cervical cancer knowledge**

	**Basu P** [32]	**Pan XF** [33 **]**	**Montgomery MP** [34]	**Current study**
Aware of cervical cancer	38.0%		15.0%	27.0%
Aware of HPV		77.9%	36.0%	13.0%
Aware of HPV vaccine	5.5%	29.0%	26.0%	6.0%
Aware of the link between HPV and cervical cancer			28.0%	7.0%
Aware of Pap smear test	18.4%		7.0%	27.0%
Had Pap smear test in the past	33.6%			33.0%
Aware of multiple pregnancies as a risk factor for cervical cancer	12.6%			1.28%
Aware of multiple sex partners as a risk factor for cervical cancer	25.8%		33.0%	2.08%
Aware of the prevention of sexually transmitted diseases prevention by condoms.			60.0%	28.0%

Table 4 clearly shows that the rates reported in our survey are remarkably lower than the previous surveys. Low levels of knowledge were also reported in a survey [27] that only 19.0% of adult Korean women knew that HPV infection was a risk factor for cervical cancer. Yet, in another study among Chinese women [28], it was found that only 26.9% of the respondents knew that HPV infections were risk factors for cervical cancer. The findings of all these studies showed that women lacked adequate knowledge of cervical cancer and risk factors. In comparison to all these studies, the current study showed relatively low levels of awareness among Uyghur women.

Similar low levels of knowledge regarding cervical cancer in the general population have also been seen in other Asian countries where there are no cervical cancer screening programs [29].

A recent survey conducted in Mangalore, India, reported that women who were between the age group of 35 to 40 years had more knowledge of cervical cancer and issues related to it [30]. This is similar to our survey that reported that the women between the age group of 31 to 40 years had more awareness of cervical cancer.

The knowledge improved significantly with improvement in the level of education. Several research studies have shown that health education through different teaching strategies is an effective way of imparting knowledge. Education is needed to prevent the incidence of cervical cancer [31,32]. The education programs are very effective in increasing cervical cancer knowledge, perceived susceptibility, and cancer prevention behaviours [15]. By education, women can be empowered with knowledge of cervical cancer, its early warning symptoms and the availability of adequate therapies [30].

A recent study conducted in Maldives reported that the exposure of the women of Maldives to the risk factors of cervical cancer is high, and the awareness about cervical cancer, its risk factors, and the methods of prevention is very limited [29]. Our survey reported the same results that despite of relatively higher incidence rate of cervical cancer in Xinjiang, less than a quarter of the respondents were not at all or very less aware of cervical cancer, HPV, and HPV vaccine.

We investigated a lowest level of knowledge or awareness, that is, having heard of HPV, lack of knowledge about cervical cancer, HPV infection, and its possible consequences are great impediment for reducing incidence of cervical cancer in high incidence area, Xinjiang.

The current national policy of providing mandatory free education to all the Uyghur women is likely to have a major impact. Thus, health literacy programs which are effective not only in increasing knowledge but also in creating a positive attitude among women towards Pap smear test should be organized to increase Pap smear coverage in Xinjiang.

## Conclusions

Overall, the study results indicated that Uyghur women’s awareness or level of knowledge and attitude regarding cervical cancer, HPV, and HPV vaccine is way too low and unsatisfactory. In order to control or reduce high incidence of cervical cancer, elevation of knowledge level about cervical cancer is more important than prophylactic vaccination in Xinjiang. Healthcare services should take serious measures to educate women about cervical cancer, HPV, and the potential value of HPV vaccination. Our data highly suggested that educational programs are needed to prevent the incidence of cervical cancer among Uyghur women of Xinjiang, a high-incidence region of cervical cancer in China. Otherwise, it will continue to be a grave health problem in Xinjiang.
